# Left main and three vessels spontaneous coronary artery dissection as an incidental finding in young man with history of Hodgkin’s lymphoma-a case report

**DOI:** 10.1186/s12872-023-03609-w

**Published:** 2024-01-03

**Authors:** Raheleh Kavyani, Soheila Salari, Zeinab Norozi, Saeid Hosseini, Saifullah Abdi, Alireza Rai, Majid Maleki

**Affiliations:** 1 Echocardiography Research Center, Rajaie Cardiovascular Medical and Research Center, Iran University of Medical Sciences, Tehran, 1995614331 Iran; 2 Rajaie Cardiovascular Medical and Research Center, School of Medicine, Iran University of Medical Sciences, Tehran, 1995614331 Iran; 3 Heart Valve Diseases Research Center, Rajaie Cardiovascular Medical and Research Center, Iran University of Medical Sciences, Tehran, 1995614331 Iran; 4 Cardiovascular Intervention Research Center, Rajaie Cardiovascular Medical and Research Center, Iran University of Medical Sciences, Tehran, 1995614331 Iran; 5 Cardiovascular Intervention Research Center, Kermanshah University of Medical Sciences, Kermanshah, 6715847141 Iran

**Keywords:** Spontaneous coronary artery dissection, Coronary artery disease, Chemotherapy, Radiotherapy

## Abstract

**Background:**

Spontaneous coronary artery dissection is a rare and important cause of myocardial infarction, especially in young women without other coronary artery disease. This arterial dissection can occur within or between any of the 3 layers.

Its predisposing factors include connective tissue diseases (Marfone syndrome, Ehlers-Danlos syndrome), vasculitis (polyarteritis nodosa, systemic lupus erythematosus, and Kawasaki disease), atherosclerosis and fibromuscular dysplasia.

Clinical presentations of spontaneous coronary artery dissection are wide spectrum from asymptomatic to acute coronary disease, sustained ventricular arrhythmia and sudden cardiac death.

**Case presentation:**

We describe A 33-year-old man with history of Hodgkin’s lymphoma five years earlier that became a candidate for Patent foramen ovale closure due to recurrent embolic cerebrovascular accident. Before the intervention, coronary angiography incidentally showed dissection in the left main and all major coronary arteries.

**Conclusions:**

Based on our hypothesis, chemoradiotherapy-induced arteriopathies could be consider as a predisposing factor for spontaneous coronary artery dissection.

**Supplementary Information:**

The online version contains supplementary material available at 10.1186/s12872-023-03609-w.

## Background

Spontaneous coronary artery dissection (SCAD) is a separation of the coronary artery wall layers that is not iatrogenic or related to trauma that recognized as an important cause of myocardial infarction (MI), especially in young women. The true incidence and prevalence of SCAD in the general population is unknown, due to significant underdiagnoses of this condition prevalence of SCAD on coronary angiography was reported to be between 0.2% to 1.1% that still seems to be underestimate [1, 2].

The arterial dissection with SCAD can occur within or between any of the 3 layers (intima, media, or adventitia) of the coronary artery wall. The dissection mostly occurs between the intima and the media but can also occur between the media and the adventitia [3, 4].

Predisposing factors for SCAD are: connective tissue diseases (Marfan syndrome, Ehlers-Danlos syndrome), vasculitis (polyarteritis nodosa, systemic lupus erythematosus, and Kawasaki disease), atherosclerosis, and fibromuscular dysplasia.

Also acute hypertension and intense emotional stress can lead to SCAD [5, 6].

Clinical presentations of SCAD are broad, almost all patients with SCAD present with Acute Coronary Syndrome (ACS) and elevation of cardiac enzymes that can complicated with ventricular arrhythmias and sudden cardiac death, but on the other hand, some patients have not any clinical symptoms [6–9].

Here in we report an asymptomatic young man with Left Main (LM) and 3 vessels SCAD as an incidental finding before patent foramen ovale (PFO) closure, based on our knowledge this extensive SCAD could be a consequence of chemoradiotherapy -induced arteriopathy and it is the first case of asymptomatic LM and 3 vessels SCAD that reported so far.

## Case presentation

A 33-year-old male with a history of (PFO) that was diagnosed after recurrent embolic cerebrovascular accident (CVA) last 2 years ago, and Hodgkin’s lymphoma five years earlier that underwent chemoradiotherapy. He was asymptomatic and had not coronary artery risk factors.

He became a candidate for PFO closure, Before the intervention, SCAD was observed as an incidental finding during diagnostic coronary artery angiography (CAG).

**Fig. 1:**
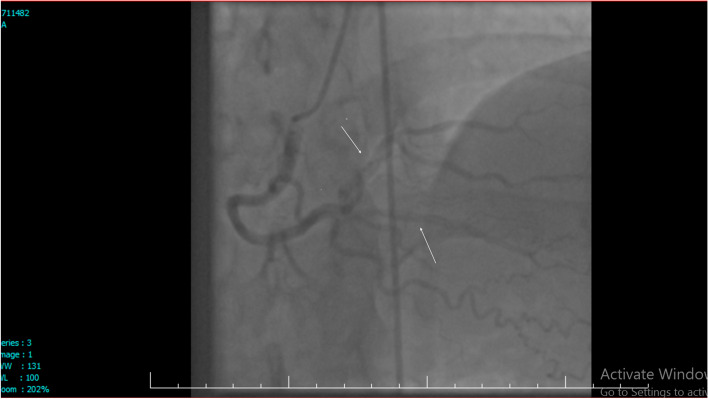
Angiography, RCA injection showed dissections in PLV

**Fig. 2:**
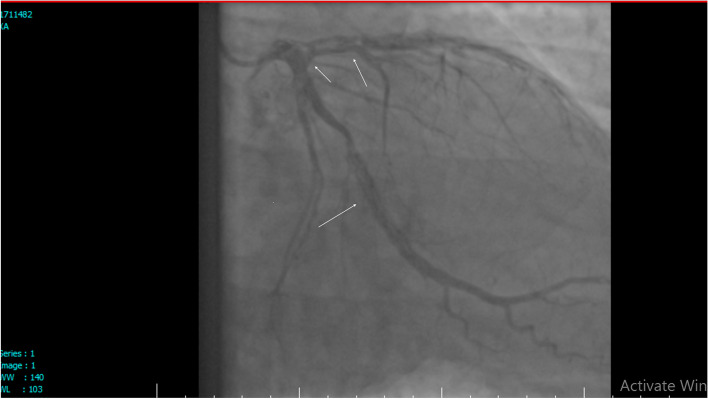
Angiography, Left coronary artery injection showed dissections in distal LM, LAD and LCX

Video 1, 2 and 3 showed SCAD in left main (LM), left anterior descending artery (LAD), right coronary artery (RCA) and also, left Circumflex artery (LCx) (Figs. 1 and 2).

Then carotid artery ultrasound was performed for evaluated the involvement of carotid and renal vessels that showed not dissection.

Since connective tissue disorder are one of the causes of SCAD and the patient had some Marfanoid appearance, genetic consultation for connective tissue diseases was consider. The patient’s genetic sequence was evaluated and there were no relevant findings also it was negative for the other connective tissue disorders.

While investigating the causes of SCAD, we found that he had mild anxiety disorders and was a professional weight lifter, which could be one of the precipitating factors for SCAD.

Since patient was asymptomatic and echocardiographic findings, electrocardiogram (ECG), level of cardiac enzymes were normal, the heart team’s decided on medical treatment. For his medication, antiplatelets and anti-lipids, beta blocker and citalopram was prescribed.

After 3 month of follow up Computed tomography angiography did not show progression in coronary artery lesions and he remained asymptomatic.

## Discussion

Pretty [1] in 1931 reported the first case of SCAD in a 42-year-old woman who died unexpectedly following repetitive retching and vomiting. which elicited coronary artery rupture from dissection of an atheromatous aneurysm [10] Although this is the first report of SCAD cited in various articles but SCAD is defined as occurring in coronary arteries without underlying disease, whereas this reported case had an atheromatous aneurysm and Seems that vomiting in this case was due to anterior MI due to cardiac event, not anterior MI due to vomiting.

SCAD is classified angiographically into 3 types. Type 1 has the classic appearance of contrast dye staining of arterial wall with multiple radiolucent lumen. Type 2 SCAD is the most common and shows long diffuse (typically > 20 to 30 mm) and smooth narrowing that varies in severity. Type 3 has focal or tubular stenosis that mimics atherosclerosis, typically requiring intracoronary imaging to prove presence of intramural hematoma or double lumen. SCAD is often underrecognized and incorrectly classified due to atherosclerosis if diagnosis is reliant upon visualization of the classic dissection flap [11, 12].

The LAD is the most frequently affected vessel accounting for 60% of coronary dissections, RCA is the second most common site (more common in males), followed by the LM, multivessel dissections in up to 20%-25% of cases, similarly in this case SCAD affected LM and 3 major coronary arteries.

Coronary dissections are more common in the mid to distal segments, often involving side branches. Eleid et al. showed that coronary artery tortuosity on coronary angiography is more common in patients with SCAD (78% vs. 17% in controls), and severe coronary tortuosity is a marker for recurrent SCAD events [13, 14].

The commonest predisposing factors in SCAD were postpartum, fibromuscular dysplasia (FMD), connective tissue disease and hormonal therapy [5, 6].

Precipitating stressors that result in a Valsalva-like activities (such as coughing, retching, vomiting) increase in thoracoabdominal pressure or that raise catecholamines can increase cardiocirculatory shear stress, which can trigger SCAD, especially in patients with underlying predisposing arteriopathies [6] This case did not have any of common underlying factors, but he was played a weight lifting that could be a potential predisposing factor.

Aother predisposing factor for SCAD is anti cancer chemotherapy agents. Cardiotoxicity is one of the most important side effects of chemotherapy. Among chemotherapy drugs, cisplatin and capecitabine (5-fluorouracil) can be mentioned as chemotherapy agents that cause a wide range of cardiovascular abnormalities such as arrhythmia or cardiopulmonary failure. Many studies point to the essential role of 5-fluorouracil in the development of vasospasm, which can be a key risk factor for SCAD. Chemotherapy based on cisplatin causes oxidative stress, reducing the activity of antioxidants and their concentration in plasma, which leads to cardiac effects in the early stages of treatment. Therefore, the use of cisplatin can cause SCAD, even when other coronary risk factors are not present [15–17].

There are studies that reports of SCAD following antineoplastic agent too. Pavel Somov et al. [18] describes a case of multivessel (LAD and LCX) SCAD, which led to acute myocardial infarction in a 24- year-old man after a course of cisplatin chemotherapy for testicular cancer. Michael B Jorgensen et al. [13] reports a 39-year-old woman who was treated with 5-fluorouracil for cervical cancer and had an ST-segment elevation myocardial infarction. Angiography showed extensive stratification of the coronary vessels, which, according to the authors, was at least partially the result of 5-fluorouracil-induced vasospasm. Another article on chemotherapy-induced arthropathy reports a case of SCAD in a 69-year-old man with colon adenocarcinoma who received four courses of chemotherapy (oxaliplatin, capecitabine) [19, 20]. The exact information about our patient’s chemotherapy regimen was not accessible.

Chest discomfort was the most common presenting symptom, as reported in 96% of patients. Less frequent symptoms include pain radiation to the arms or neck, nausea or vomiting, diaphoresis, dyspnea, and back pain. Although some of these presentation, like vomiting could be a presentation of acute MI, not a predisposing factor. In overall, 34% of SCAD patients had unstable clinical conditions such as (ongoing pain and/or ischemia or stuttering and/or recurrent that required medications for pain relief before arrival at the catheterization laboratory [6, 9].

Not only exact prevalence of SCAD is clear, accordingly, prevalence of asymptomatic SCAD is not clear too. There are scanty case reports of SCAD patients who were completely asymptomatic and the diagnosis of SCAD is made as an incidental finding months or years after its occurrence on coronary angiography or CT angiography. Habib A et al. [21] reports a 2 case of SCAD that were asymptomatic and patients diagnosis during evaluation before non-cardiac elective surgery.The first case was a 48-year-old woman was admitted to gynecological surgery base on her abnormal baseline ECG she was referred for an exercise echocardiogram which showed evidence of ischemia in the anteroapical wall and coronary angiography revealed a localized dissection in the proximal LAD. And the other case was A 40-year-old man with history of non-small cell lung cancer and was scheduled for surgical resection. Nuclear image showed a fixed perfusion defect in the apical septum, therefore underwent coronary angiography that showed an extensive dissection originating in the (LAD) and extending to the distal LAD and the diagonal branch. These patients had not known predisposing factor for SCAD.

We present a case of SCAD that we believe is the first report of SCAD involving the LM and all major coronary arteries and branches. Despite extensive coronary artery involvement, he was asymptomatic and had no ECG changes or positive cardiac enzymes. Echocardiography was normal and there were no common predisposing factors except weight bearing, which to our knowledge could not justify this extensive dissection.

Considering the history of Hodgkin’s lymphoma, our hypothesis about the predisposing factor of SCAD is arteriopathy caused by chemoradiotherapy, that with weight lifting they could be the precipitating factors could explain this wide dissection.

Optimal treatment strategies for SCAD remains undetermined and the guidelines for ACS have not been specifically recommended for SCAD then a current recommendations comes from the case reports and retrospective reviews.

Conservative management is preferred in stable patients with SCAD as most dissected segments will heal spontaneously. Medical therapy is based upon opinion, with no randomized clinical trials in this area.

In SCAD cases similar to standard ACS treatment with the dual antiplatelet agents, heparin and beta-blockers are preferred strategies, Glycoprotein IIb/IIIa inhibitors have also been used without complications [20]. That’s why our case underwent Conservative management with standard ACS treatment.

However, these agents could potentially delay healing of the intramural hematoma and lead to dissection extension intravenous fibrinolysis should not be used as it has the potential to exacerbate the dissection or hematoma, worsen the condition, and even result in mortality [20].

If the patient is hemodynamically unstable and have sustained ventricular arrhythmias, complete vessel occlusion which is unlikely to resolve with medical treatment alone, LM involvement, ongoing ischemia or recurrent chest pain, Percutaneous coronary intervention (PCI) or coronary artery bypass grafting (CABG) may be preferred [22, 23] but is associated with significant challenges.

Recurrent SCAD is an important complication in SCAD patients and was defined as de novo recurrent spontaneous dissection with new recurrent MI symptoms and enzyme elevation, which did not involve extension of dissection of the original SCAD lesion [24].

One of the condition that significantly associated with an increased risk of recurrent SCAD is hypertension, while the use of beta-blockers was significantly associated with reduction in the risk of recurrent SCAD. Then beta-blockers are recommended in all patients, with the potential to reduce arterial shear stress, facilitate healing and reduce long-term recurrence [25].

In this case our concern about the recurrence of dissection is related to underlying arteriopathy, the extensive dissections and his treatment strategy. Therefore regular follow-up is required to continuation of drug treatments such as beta-blockers, avoiding stressful conditions and heavy physical activities associated with the valsalva maneuver (forget weightlifting). He remains asymptomatic and uneventful during 12 month close follow up.

## Conclusion

SCAD is a rare but an important cause of MI. Base on this special report, arteriopathies due to chemoradiotherapy could be consider as a predisposing factor especially in the pattern of extensive SCAD like our case in LM and three coronary arteries.

### Supplementary Information

**Additional file 1: Video 1.** Right coronary system injection showed: Dissection in PDA (posterior descending artery). https://uupload.ir/view/video_1_t084.mp4.

**Additional file 2: Video 2.** Right coronary system injection showed: Dissection in PLV. https://uupload.ir/view/video_2_izxa.mp4.

**Additional file 3: Video 3.** Left coronary system injection showed: dissection in LM, LAD that extend to distal and all branches of LAD and LCX. https://uupload.ir/view/video_3_f6o8.mp4.

## Data Availability

The datasets used and/or analyzed during the current study are available from the corresponding author on reasonable request.

## Abbreviations

PCI: Percutaneous coronary intervention
CABG: Coronary artery bypass grafting
TIMI: Thrombolysis In myocardial infarction
MI: Myocardial ischemia
FMD: Fibromuscular dysplasia
PFO: Patent foramen oval
SACD: Spontaneous coronary artery dissection
ACS: Acute coronary syndrome
LM: Left main
RCA: Right coronary artry
LAD: Left anterior descending
LCX: Left circumflex
PLV: Posterior left ventricular artery
